# Diagnostic performance of oral swab specimen for SARS-CoV-2 detection with rapid point-of-care lateral flow antigen test

**DOI:** 10.1038/s41598-022-11284-8

**Published:** 2022-05-05

**Authors:** Arati Mane, Shilpa Jain, Ankita Jain, Michael Pereira, Atul Sirsat, Gaurav Pathak, Vikalp Bhoi, Shailaja Bhavsar, Samiran Panda

**Affiliations:** 1 Division of Microbiology, ICMR-National AIDS Research Institute, 73, G block, MIDC, Bhosari, Pune, Maharashtra India; 2 Old Bhosari Hospital, Bhosari, Pune, Maharashtra India; 3 Division of Epidemiology and Communicable Diseases, Indian Council of Medical Research, New Delhi, India

**Keywords:** SARS-CoV-2, Infectious-disease diagnostics

## Abstract

We evaluated the performance of oral swab specimen both health-care worker (HCW) collected and self-collected for severe acute respiratory syndrome coronavirus-2 (SARS-CoV-2) detection with rapid antigen test (RAT) as compared to reverse transcriptase polymerase chain reaction (RT-PCR). Of the 529 participants enrolled, 121 (22.8%) were RT-PCR positive. Among the RT-PCR positives, 62 (51.2%) were RAT positive using oral swab. When compared with RT-PCR, RAT with oral swab had sensitivity and specificity of 63.3 and 96.8% respectively among symptomatic individuals. No statistically significant difference was observed in RAT positivity with HCW collection and self-collection, *p* = 0.606. Ct values were significantly lower in RT-PCR and RAT positive samples (ORF gene: 18.85 ± 4.36; E gene: 18.72 ± 4.84) as compared to RT-PCR positive and RAT negative samples (ORF gene: 26.98 ± 7.09; E gene: 26.97 ± 7.07), *p* < 0.0001. Our study demonstrated moderate sensitivity of RAT with oral swab in symptomatic individuals. Oral swab was the preferred sampling by almost all participants in terms of convenience and comfort as compared to nasopharyngeal swab. Oral swabs have utility for SARS-CoV-2 antigen detection among symptomatic individuals residing in remote rural areas and can serve as an initial screening tool during COVID-19 spikes when cases rise exponentially and laboratory capacities for RT-PCR testing become overwhelmed.

## Introduction

The spread of severe acute respiratory syndrome coronavirus 2 (SARS-CoV-2), the causative agent of the coronavirus disease 2019 (COVID-19) surpassed many predictions and has created an evolving global public health and economic crisis^1–3^. Early identification of SARS-CoV-2 infected individuals and treatment in isolation is recommended as the key intervention measure to reduce community transmission and preventing future spikes. With efforts being made to get back life to normal by graded opening of work places, colleges/schools and eateries, there is a need to observe caution. This can be achieved through availability of easy screening tools and using them for quickly arriving at intervention decisions in case of identification of clusters of suspected cases of symptomatic COVID-19.

Reverse transcriptase polymerase chain reaction (RT-PCR) is the gold standard for detection of SARS-CoV-2 infection^4,5^. However, it is a resource intensive technique in terms of requirement of laboratory set-up and workforce. On the other hand, rapid antigen tests (RAT) serve as useful screening tools for SARS-CoV-2 infection with their ease-of-use, speed of result and low cost^6–9^. However, most RAT use nasopharyngeal (NP) swabs for testing that requires travelling to designated facilities, trained health care workers (HCW) for specimen collection and individuals experience discomfort and pain during the collection procedure. NP sampling is also difficult in pediatric and geriatric populations and in individuals with altered mental status or nasal trauma/anomalies^10,11^.

Search for non-NP sampling alternatives for SARS-CoV-2 detection is thus ongoing. Nasal swabs are now approved for use with RAT that enable self-collection and self-testing at home. Likewise evaluation of saliva for SARS-CoV-2 antigen detection is being reported^12,13^. However, no literature is available regarding the performance of oral swab for SARS-CoV-2 detection with RAT. Oral swabs, if found to perform comparable to NP swabs, will offer a further simpler sampling alternative and enhance self-testing. The present study was conducted to determine the performance of oral swab specimen for SARS-CoV-2 detection with rapid point-of-care lateral flow antigen test compared to RT-PCR.

## Methods

The study was reviewed and approved by the Institutional Ethics Committee of Indian Council of Medical Research-National AIDS Research Institute (ICMR-NARI), Pune, India [NARI/EC/Approval/2021/484]. The Scientific Advisory Committee (SAC) of ICMR-NARI provided necessary clearance for implementation of the study. The study participants provided written informed consent and all methods were carried out in accordance with approved guidelines and relevant regulations.

### Study setting and participants

In this cross-sectional study, consecutive COVID-19 facility attendees at the Old Bhosari Hospital (OBH), Pune, above 18 years of age with symptoms of suspected SARS-CoV-2 infection or as asymptomatic high-risk contacts of COVID-19 cases were invited for participation. Individuals, providing written informed consent were enrolled. Participants were excluded if either of the swab specimens for RAT or RT-PCR could not be collected. Socio-demographic information, clinical symptoms and COVID-19 vaccination status of the participants were collected via structured questionnaire. Participants’ preference about sample collection procedure and experience while samples were being collected from them were taken. Feedback from HCWs involved with oral swab collection and RAT testing were obtained as well.

### Study phases and sample collection

**Figure 1:**
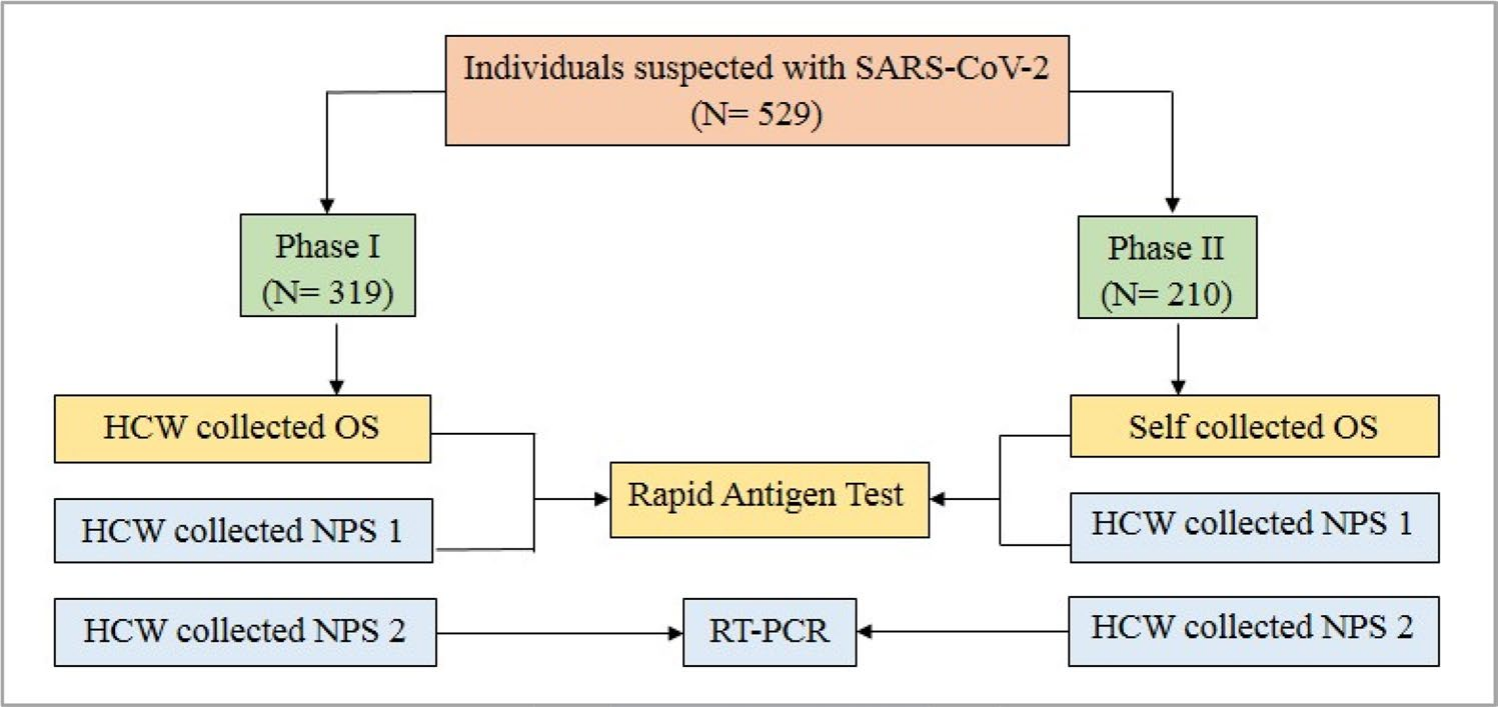
Work flow for the Study. HCW: Health Care Worker; OS: Oral Swab; NPS: Nasopharyngeal Swab.

The study was conducted from 19th June to 1st July 2021 in two phases. In Phase I, oral swabs were collected by HCW (trained dentists collected samples at the OBH COVID-19 facility), while in Phase II, oral swabs were self-collected by the participants; flocked nylon swabs were used. The workflow of specimen collection and testing is shown in Fig. 1. In the first phase, HCWs rubbed the tip of the swab on the dorsal and ventral surface of the tongue and the buccal mucosal surface of each cheek for 10 s. The swab was put in the buffer tube provided with the RAT kit. In the second phase, participants were provided with pictorial pamphlets illustrating the procedure for oral swab collection. Each participant self-collected the oral swab and put in the buffer tube provided with the RAT kit.

In both phases of the present investigation, HCWs collected two NP swab specimens from each participant, one for RAT and the other for RT-PCR (along with an oropharyngeal swab in 3 ml viral transport medium) as per standard procedure which were transported to ICMR-NARI COVID-19 laboratory for RT-PCR testing^14^.

### Sample processing

RAT was performed on-site directly after sampling. RAT used in the study was Coviself, Pathocatch (Mylab Discovery Solutions Pvt. Ltd., Maharashtra, India), a qualitative immunochromatographic test for detection of SARS-CoV-2 specific antigens. This kit is validated by ICMR for use with NP and nasal swabs. The test procedure followed in the present investigation with oral swab was as per manufacturer’s instructions. The visual read-outs were available within 20 min of the test.

For SARS-CoV-2 RT-PCR, viral RNA was isolated from the VTM using the MDS Viral RNA Extraction kit (MetaDesign Solutions, Gurgaon, India) and tested for SARS-CoV-2 with the Covidsure Multiplex RT-PCR kit (Trivitron Healthcare Labsystems Diagnostics, Chennai, India) on the CFX96 Real-Time Detection System (Bio-Rad, Hercules, CA, USA). The kit targets the E and ORF genes of SARS-CoV-2 and uses RPP30 human gene as internal control. The test was considered positive if cycle threshold (Ct) value was less than 35 for the genes tested.

Staff performing RAT were blinded to the results of RT-PCR tests and vice versa.

### Statistical analysis

Descriptive statistics are presented as means for continuous and as proportions for categorical variables. The independent t-test or Mann–Whitney test was applied to continuous variables and Fisher’s exact test to the categorical variables for comparisons. RAT results of oral swab were compared to RT-PCR and sensitivity, specificity, positive predictive value (PPV) and negative predictive value (NPV) with 95% confidence intervals (95% CI) were calculated. Statistical analysis was done using GraphPad statistical software (GraphPad Software Inc, USA) and MedCalc statistical calculators (https://www.medcalc.org/calc/). *p* value of < 0.05 was considered to be indicative of statistical significance.

## Results

### Participant characteristics

A total of 529 participants were enrolled in the study, 319 in Phase I where oral swabs were collected by HCWs and 210 in phase 2 where oral swabs were self-collected. The participant characteristics are presented in Table 1. No significant difference was noticed between individuals participating in both phases of the study.

**Table 1:** Characteristics of study participants.

Characteristic	Phase I Healthcare worker collection (N = 319)	Phase II Self Collection (N = 210)	*p*
Age in years (mean ± SD)	30.6 (10.9)	29.5 (10.3)	0.246
**Sex** **N (%)**			
Male	251 (78.7)	153 (48.0)	0.142
Female	68 (21.3)	57 (17.9)	
**Symptomatic** **N (%)**			
Yes	109 (34.2)	63 (19.7)	0.343
No	210 (65.8)	147 (46.1)	
Days since symptom onset (mean ± SD)	2.4 ± 1.4	2.6 ± 1.5	0.380
**COVID-19 vaccine** **N (%)**			
Taken	45 (14.1)	33 (10.3)	0.618
Not taken	274 (85.9)	177 (55.5)	

### RT-PCR and RAT with NP swabs

Of the total 529 participants, 121 (22.8%) tested positive by RT-PCR (73/319, 22.8% during Phase I and 48/210, 22.9% during Phase II). Among the RT-PCR positives, 81 (66.9%) tested positive by NP RAT, with sensitivity and specificity of 87.3% (77.9–93.8) and 100% (96.1–100) respectively among symptomatic individuals and 28.6% (15.7–44.6) and 100% (98.8–100) respectively among asymptomatic individuals.

### Diagnostic performance of RAT with oral swab

Among the RT-PCR positives, 62 (51.2%) tested positive by oral swab RAT (36/73, 49.3% during Phase I and 26/48, 54.1% during Phase II). There was no statistically significant difference in RAT positivity with sample collection by HCW and self-collection, *p* = 0.606. No invalid results with RAT were observed.

Among symptomatic individuals, RAT had sensitivity, specificity, PPV and NPV of 63.8% (48.5–77.3), 96.8% (88.8–99.6), 93.8% (79.1–98.4) and 77.9% (70.7–83.8) respectively with HCW mediated oral swab collection and 62.5% (43.7–78.9), 96.7% (82.8–99.9), 90.9% (71.9–97.5) and 70.7% (60.5 to 79.2) respectively with self-collection while compared with RT PCR (Table 2).

**Table 2:** Comparison of performance of Rapid Antigen Test with Oral Swab in symptomatic individuals: HCW *versus* self-collection.

RT-PCR					RT-PCR				
Oral RAT		Positive	Negative		Oral RAT		Positive	Negative	
(HCW collection)	Positive	30	2	Sensitivity: 63.8% Specificity: 96.8%	(Self-collection)	Positive	20	1	Sensitivity: 62.5% Specificity: 96.7%
	Negative	17	60			Negative	12	30	
	Total	47	62			Total	32	31	

*RAT* Rapid antigen test, *RT-PCR* Reverse transcriptase-polymerase chain reaction, *HCW* Health care worker.

Among asymptomatic individuals, RAT had sensitivity, specificity, PPV and NPV of 23.1% (8.9–43.6), 96.8% (93.3–98.8), 50% (25.8–74.2) and 89.9% (87.8–91.7) with HCW mediated oral swab collection and 37.5% (15.2–64.6), 98.5% (94.6–99.8), 75% (39.8–93.2) and 92.8% (89.82–94.9) with self-collection respectively when compared with RT PCR.

### RAT positivity with oral swab by Ct values

RAT results stratified by Ct value cut-offs are presented in Table 3.

**Table 3:** Oral swab Rapid Antigen Test results by Cycle threshold (Ct) value cut-offs.

Ct value (ORF gene)	Total N (% within total)	Rapid antigen test positives N (% within Ct value)
≤ 25	80 (66.1)	57 (71.3)
> 25	41 (33.9)	05 (12.2)
≤ 30	88 (72.7)	59 (67.04)
> 30	33 (27.3)	03 (9.1)
Total	121 (100)	62 (51.2)

**Figure 2:**
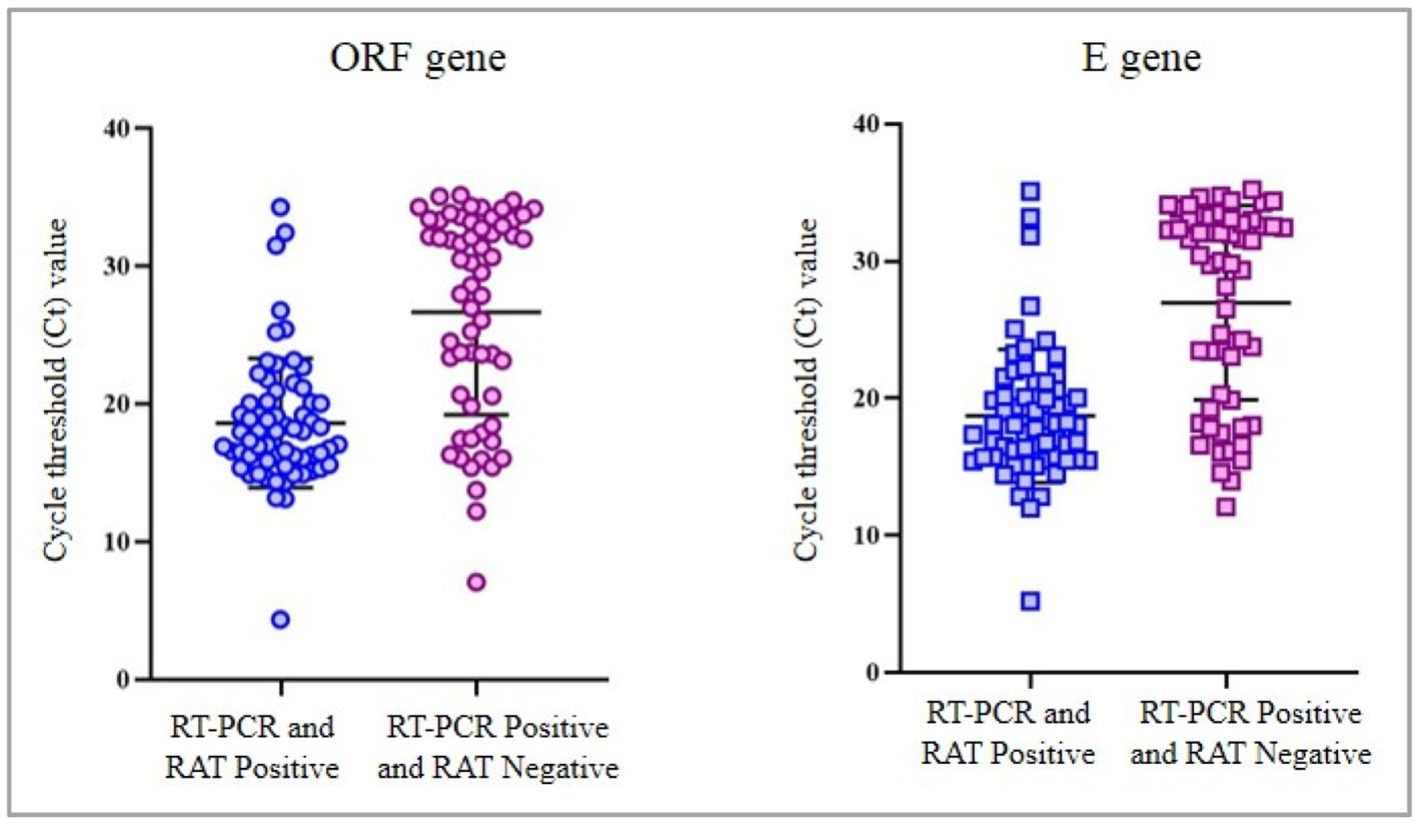
Cycle threshold values in Rapid Antigen Test positive and negative samples. RAT: Rapid Antigen Test; RT-PCR: Reverse Transcriptase-Polymerase Chain Reaction.

The Ct values were significantly lower in RT-PCR positive and RAT positive samples (ORF gene: 18.85 ± 4.36; E gene: 18.72 ± 4.84) as compared to RT-PCR positive and RAT negative samples (ORF gene: 26.98 ± 7.09; E gene: 26.97 ± 7.07), *p* < 0.0001 (Fig. 2). We observed a trend of decreasing RAT positivity with increasing Ct values was observed, *p* < 0.0001.

### RAT positivity with oral swab by duration of symptoms

**Figure 3:**
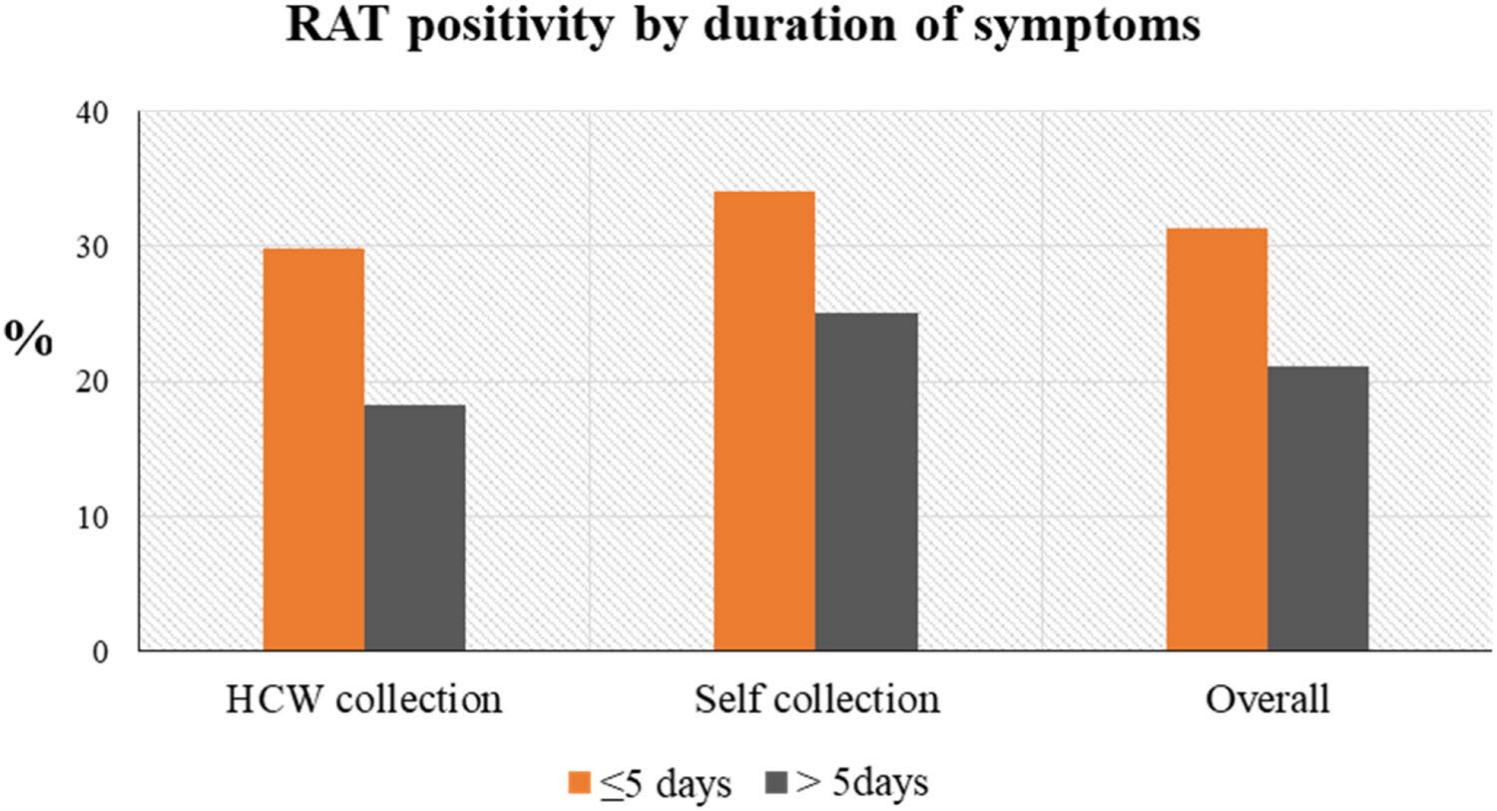
Rapid Antigen Test positivity (RAT) by duration of symptoms.

RAT positivity by duration of symptoms (in days) with self-collection and HCW collected oral swabs is presented in Fig. 3. COVID-19 duration of ≤ 5 days corresponded to RAT positivity rate of 31.3%, while COVID-19 duration of more than 5 days had RAT positivity rate of 21.1%.

### Feedback from participants and HCW with regards to oral swab sampling

Almost all participants (524/529, 99.1%) chose oral swab as the most convenient and comfortable sample for SARS-CoV-2 testing as compared to NP sampling. We collected feedback from the HCW involved with oral swab collection. HCW opined that patients were more co-operative during oral swab collection compared to NP swab. There was no coughing and sneezing during the sample collection procedure. During the phase 2 of the study, the participants were willing for self-collection and followed the instructions in the pamphlet carefully. The drawback with regards to oral RAT testing as told by HCW were that, while performing the test the material from the swab at times settled at bottom of the buffer tube leading to delay in flow and that the readings have to be taken meticulously as some positive results appeared as light bands.

## Discussion

Easy sampling alternatives will enhance the uptake of rapid antigen testing and accelerate COVID-19 detection. Oral swabs have been previously used for detection of infectious etiologies^15–17^ and disease biomarkers^18,19^, though, their performance for SARS-CoV-2 detection with RAT has not been reported. In the present study we evaluated the performance of oral swab specimen for detection of SARS-CoV-2 antigen. Similar results were obtained for RAT with HCW collected and self-collected oral swabs, an observation that supports self-sampling^10,20–22^_**.**_

When compared with RT-PCR, RAT with oral swab sampling had a moderate sensitivity of 63.3% among symptomatic individuals, which was reduced to 28.6% among asymptomatic individuals. The sensitivity of RAT is influenced by the viral load in the specimen as evidenced by decrease in RAT positivity with increasing Ct values. RAT performed with oral swab detected 71.3% cases with Ct values < 25 that are considered to be highly infectious. However, our data also revealed that some individuals with low Ct values were missed by RAT. Hence, RAT with oral swab sampling cannot be a reliable replacement for SARS-CoV-2 detection.

The performance of RAT varies by number of factors, primarily, the clinical status of the patient (symptomatic versus asymptomatic), the RAT kit used, the specimen type used and time of testing (fresh versus stored specimen). NP swab is the recommended specimen for most RAT kits. A systematic review and meta-analysis conducted for the accuracy of SARS-CoV-2 rapid antigen test kits reported a pooled sensitivity of 76.3% with NP swabs^23^. However, NP swab collection is invasive, limited by need of a trained HCW for sample collection and poses risk of infection transmission to HCW. Among the alternative samplings, the sensitivity reported with anterior nasal/mid-turbinate swabs (75.5%) is comparable with NP swabs, while comparatively lower sensitivities with oropharyngeal (53.1%) and saliva (37.9%) specimens are reported^23^. Currently there is no literature with regards to use of oral swab for SARS-CoV-2 antigen detection.

NP swabs will remain as favored approach for testing with RAT. The oral swab with its ability to detect majority of the infectious cases, will have utility for testing among symptomatic individuals, especially for those residing in remote rural areas without the availability of trained HCWs and RT-PCR testing facilities. In such settings, conducting RAT test with self-administered oral swab will have the potential to enable detection and timely isolation of SARS-CoV-2 infected individuals in order to forestall local spread of infection. Oral swab also has the potential to serve as an initial screening tool in situations of COVID-19 surge when the cases rise exponentially and testing with RT-PCR becomes overwhelming for the laboratories. However, development of additional strategies to minimize false negative results will have to be laid down in these settings. These can include referral of individuals with symptoms compatible with COVID-19 but a negative antigen result for RT-PCR testing.

RAT with oral swab sampling had an overall specificity of 97.3%. We acknowledge that the RAT kit used in the study was designed for testing with NP and nasal swabs. As per the experience of HCWs, the viscous material from oral swab at times settled at the bottom of the buffer tube and false positive results were particularly observed in these samples. Similar observation was made by Chaimayo et al. while evaluating the rapid Standard Q COVID Ag test with respiratory specimens^24^.

Our study had strengths and limitations. It was conducted in a real world setting and included both symptomatic and asymptomatic individuals. We demonstrated participants’ acceptability for oral swab and the feasibility of its use for RAT testing. However, we did not include pediatric age-group and individuals with nasal anomalies, who we feel could have benefited more with this sampling approach, warranting further studies in these population groups and using different RAT kits. One time antigen test may miss a few positive cases, while serial testing will identify individuals who are likely to become infectious in the following few days due to viral proliferation and viral loads go high enough to be detected by RAT, specifically among asymptomatic individuals^25,26^. Thus, future studies should incorporate a component of serial testing to see whether it can compensate for the lower sensitivity observed.

To conclude, our study demonstrated moderate sensitivity of oral swab specimen for SARS-CoV-2 detection antigen detection in symptomatic individuals. Oral swab, was the preferred sampling by almost all participants in terms of convenience and comfort as compared to NP swab. Oral swabs will have utility for SARS-CoV-2 antigen detection among symptomatic individuals residing in remote rural areas and can also serve as an initial screening tool during COVID-19 spikes when cases rise exponentially and laboratory capacities for RT-PCR testing become overwhelmed.
